# A Review of Behavioral Observation Coding Approaches for the Trier Social Stress Test for Children

**DOI:** 10.3389/fpsyg.2018.02610

**Published:** 2018-12-21

**Authors:** Kristel Thomassin, Jacquelyn Raftery-Helmer, Jacqueline Hersh

**Affiliations:** ^1^Department of psychology, University of Guelph, Guelph, ON, Canada; ^2^Department of psychology, Worcester State University, Worcester, MA, United States; ^3^Department of psychology, Appalachian State University, Boone, NC, United States

**Keywords:** Trier Social Stress Test, children and adolescents, review, observational methods, stress response

## Abstract

The Trier Social Stress Test (TSST) has become one of the most widely-used protocols for inducing moderate psychosocial stress in laboratory settings. Observational coding has been used to measure a range of behavioral responses to the TSST including performance, reactions to the task, and markers of stress induced by the task, with clear advantages given increased objectivity of observational measurement over self-report measures. The current review systematically examined all TSST and TSST-related studies with children and adolescents published since the original work of Kirschbaum et al. (1993) to identify behavioral observation coding approaches for the TSST. The search resulted in 29 published articles, dissertations, and master's theses with a wide range of coding approaches used. The take-home finding from the current review is that there is no standard way to code the Trier Social Stress Test for Children (TSST-C), which appears to stem from the uniqueness of investigators' research questions and sample demographics. This lack of standardization prohibits conclusive comparisons between studies and samples. We discuss relevant implications and offer suggestions for future research.

## Introduction

The Trier Social Stress Test (TSST; Kirschbaum et al., 1993) has become one of the most widely-used protocols for inducing moderate psychosocial stress in laboratory settings. Highly standardized, the TSST consists of an anticipation period followed by a test period during which participants deliver a speech as if they are at a job interview and perform mental arithmetic in front of a panel of “experts” or “judges,” who are experiment confederates trained to appear stoic. Participants are informed their performance will be evaluated by the panel of experts, which is designed to induce stress as participants anticipate possible negative judgements regarding their performance. The TSST protocol contains all the elements of a stress-inducing task, including a threat to the social self, uncontrollability, and unpredictability (Dickerson and Kemeny, 2004).

Although it was originally developed for use with adult samples, the TSST has since been modified for children (TSST-C) and has been used with children as young as 7 years old (e.g., Buske-Kirschbaum et al., 1997, 2003). To further accommodate a range of subsamples, developmental levels, and experimental constraints, various other modifications have been used such as omitting the post-speech arithmetic task altogether (e.g., Heilbron et al., 2008), modifying the duration of the preparation and delivery phase of the speech (e.g., Jordan, 2008; Niekerk et al., 2017), and using video-recording rather than live judges (e.g., Cartwright-Hatton et al., 2003). In addition, studies diverge in the content or topic of the speech and common alterations have included asking participants to present on how a story would unfold (e.g., Panjwani et al., 2016; Wedl et al., 2016), the content of a text (e.g., Roth and Herzberg, 2017), one of multiple provided topics (Oppenheimer et al., 2016), running for class president (Geiss, 2016), and what makes a good friend (Benoit, 2013; Rith-Najarian et al., 2014).

The TSST has become the standard protocol for experimentally inducing psychosocial stress in participants, and studies have examined a range of outcomes in response to the task, including biological parameters and subjective reports of stress (Kudielka et al., 2007). Compared to other laboratory stressors, the TSST has demonstrated the most consistent associations with physiological markers of stress; it has been shown to reliably induce hypothalamus-pituitary-adrenal (HPA) and cardiovascular responses (Dickerson and Kemeny, 2004). To date, there is no evidence for age differences in stress responses to the TSST (Kudielka et al., 2004), and consistent with its use among adults, it reliably elicits both autonomic nervous system and HPA axis reactivity in adolescents (Kudielka et al., 2007; Gunnar et al., 2009; Stroud et al., 2009). The TSST also lends itself to subjective reports of stress, anxiety, and performance, which youth typically report using Likert-type scales (e.g., the PANAS-C; Laurent et al., 1999; the Self-Assessment Manikin Scale; SAM; Buse et al., 2016); however, subjective reports come with a range of biases, particularly for younger children.

Observational coding has been used to measure a range of behavioral responses to the TSST, including performance, reactions to the task, and markers of stress induced by the task, and offers a more objective measurement approach (e.g., Rith-Najarian et al., 2014). There are clear advantages to utilizing observational coding, more generally, and specifically for the TSST, given research showing that children's self-report of emotions, such as the anxiety they may experience while preparing for a speech, do not fully capture their experience (Casey, 1993; Hubbard et al., 2004). For example, using observational measures during the TSST may more accurately assess external expression of emotion or behavior during stress, especially as compared to self-report or physiological assessment tools that may only capture children's internal emotional experiences (Denham, 1998; Eisenberg and Fabes, 1999). Despite this, there is no standardized coding scheme for the TSST, requiring researchers to adapt existing coding schemes or develop their own (Rith-Najarian et al., 2014).

The current review systematically examined all TSST and TSST-related studies with children and adolescents published since the original work of Kirschbaum et al. (1993) with the goal of identifying the myriad of ways that performance on the TSST has been measured. In particular, we aimed to compile existing behavioral observation coding approaches so as to provide guidance and recommendations for researchers seeking to measure TSST performance via objective coding.

## Method

### Search Strategy

We were interested in behavioral observation coding approaches to the TSST or any speech stressor task. Therefore, keywords used for this search included: “trier social stress test,” “social stress test,” “social stress task,” “psychosocial stressor,” and “speech task.” Even though the target sample was children or adolescents, these keywords were not included at this stage to make sure no article was overlooked. Instead, this inclusionary criterion was examined at the article review stage (see below). All keywords were searched in the following databases: ERIC, Medline, PsycINFO, PQDT, Scopus, and Web of Science. Articles published after the original paper describing the TSST (Kirschbaum et al., 1993) up to April 2017 were included. All articles were required to meet the inclusion and exclusion criteria listed below.

### Inclusion and Exclusion Criteria

1. The article included the TSST, a modified version of the TSST, or a speech task that included a social stress component, such as preparing a speech and presenting it in front of judges or a camera.
2. The sample included children and/or adolescents (aged <18 years old).
3. The methodology included a coding approach of some aspect of TSST speech delivery (e.g., facial expressions, quality of speech, gaze, emotion, etc.)

Excluded studies were ones that included a sample of adults only aged 18 or over and studies that examined speech performance or quality using interview or questionnaire methods. We also excluded studies that focused on pre- or post-speech delivery coding. For a comprehensive overview, we included dissertations and master's theses.

### Review Approach

**Figure 1:**
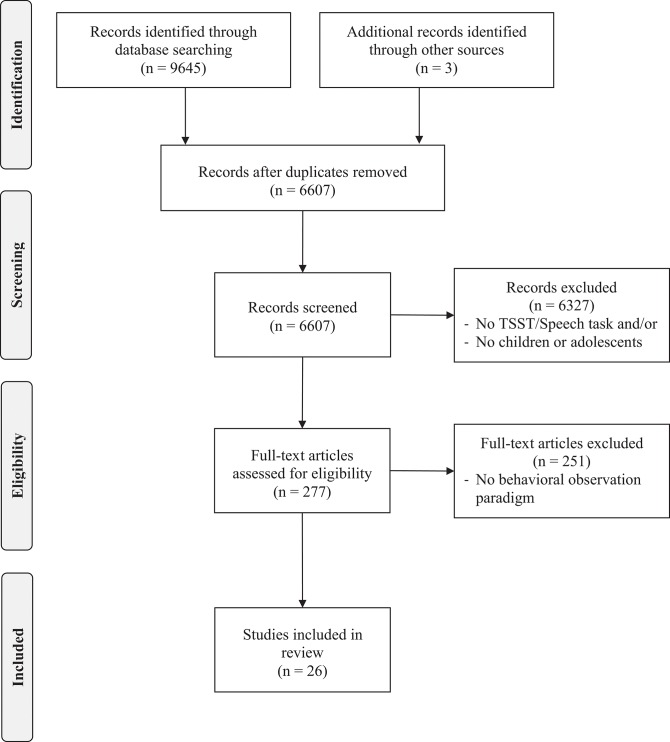
PRISMA flow diagram for the different phases of the systematic review.

The review of articles was conducted in three waves. First, all titles and abstracts (*N* = 6,603) were examined by two coders to determine (1) whether they included a TSST or speech task and (2) whether the sample included children and/or adolescents. This resulted in 277 articles. Then, articles were examined by two coders for the inclusion of any behavioral observation paradigm, leaving 26 articles. We examined the references sections of articles and found two additional articles (Cartwright-Hatton et al., 2003; Miers et al., 2009). We also emailed known authors for unpublished papers leading us to one unpublished dissertation (Lau, 2017). Finally, the remaining articles were reviewed more closely by the first and second authors and coded for type of behavioral observation paradigm used. When insufficient information was provided, we contacted the authors for their coding scheme. The final number of articles included in this review was 29 (see Figure 1).

## Results

### Descriptive Characteristics

Of the 29 studies reviewed, four were dissertations (Jordan, 2008; Benoit, 2013; Lievesley, 2014; Lau, 2017) and one was a master's thesis (Lanteigne, 2011). Eight used the TSST for children, and five included a psychosocial speech stressor task, but without the mathematical operation after the speech. Fourteen other articles were also included because they included a modified speech task (e.g., a speech stressor task with video-recording instead of live judges). Of the articles reviewed, 13 included samples of children (ages 7–12), seven included samples of adolescents (ages 13–19), and 9 included mixed samples of children and adolescents. Only 3 coding schemes, the Performance Questionnaire—Observer, the Social Performance Rating Scale, and the coding scheme used by Beetz et al. (2011, 2012), was published more than once. In total, we found 24 unique coding approaches, resulting in significant heterogeneity. The studies varied in the number of codes they examined, the type of coding they used (e.g., global, interval, event-based coding approaches), and how they used the codes to answer research questions. Below, we review the main constructs examined by each article. The primary coding approaches are focused on non-verbal behavior, emotion, and global performance. We also review other coding approaches such as those focused on dyadic and coding of tics and social skills. Articles reviewed are summarized in Table 1.

**Table 1:** List and descriptive characteristics of studies reviewed.

**References**	**Type of sample**	**Age range of sample**	**Stressor task**	**Overview of coding components**	**Final coding variables (reliability)**
Beetz et al., 2011	School (*N* = 88 males) *Subsample of children with insecure/disorganized attachment (n = 31) randomized to 1 of 3 social supporter conditions*	7–12	Modified TSST-C *Presence of a social supporter*	Other (dyadic)	A total of 49 codes were assessed Variable tested (*n* = 8): frequency and duration of talking to the social supporter; body contact with social supporter; stroking/petting social supporter; holding social supporter (Reliability not reported)
Beetz et al., 2012	School (*N* = 88 males) *Subsample of children with insecure/disorganized attachment (n = 47) randomized to 1 of 3 social supporter conditions*	7–11	Modified TSST-C *Presence of a social supporter*	Other (dyadic)	A total of 49 codes were assessed Variable tested: percent of physical contact with social supporter (Reliability not reported)
Benoit, 2013 (Dissertation)	Mixed (*N* = 55) *Anxiety* (*n* = 37) *PTSD* (*n* = 1) *No anxiety diagnosis* (*n* = 17)	7–12	Modified speech task *Speech delivered to one-way mirror* *Participants told that videotapes of their speeches would be evaluated by peers*	Emotion Non-verbal Behavior	Individual codes: Anxiety; avoidance; non-compliance; engagement; number of prompts given by experimenter; quality of content; quality of presentation style Avoidance Composite: avoidance, non-compliance, and engagement (Inter-rater reliability: ICC = 0.83 for anxiety, 0.40 for non-compliance, 0.89 for avoidance, 0.82 for engagement, 0.81 for content, and 0.71 for style)
Blöte et al., 2015	Community *High social anxiety (N = 20)* *Low social anxiety (N = 20)*	11–19	Leiden Public Speaking Task *Speech delivered to pre-recorded audience*	Emotion Non-verbal Behavior	Speech Performance Observation Scale for Youth (SPOSY): Expressiveness; lack of confidence; agitation (Internal consistency: α = 0.92 for expressiveness, 0.78 for lack of confidence, and 0.70 for agitation; Inter-rater reliability: ICCs = 0.92, 0.85, and 0.87 for expressiveness, lack of confidence, and agitation, respectively)
Borelli et al., 2017	Community (*N* = 34)	Age range not provided. *M* = 11.97 ± 1.97	Modified speech task *Participants delivered speech their mother and two judges or three strangers*	Emotion	Non-verbal anxiety cues (Inter-rater reliability: ICC = 0.79)
Burkholder et al., 2016	Community (*N* = 161)	9–10, 15–16	TSST-C	Emotion Non-verbal Behavior	Child and Adolescent Stress and Emotion Scale (CASES): Bodily, vocal, and facial signs of positive emotion, sadness/worry, anger/frustration, and anxiety (Inter-rater reliability: ICC = 0.80 for anxiety)
Buse et al., 2016	Clinical *Tic Disorder* (*N* = 31)	7–17	Speech task	Other (tics)	Frequency of tics (Inter-rater reliability = 80% agreement)
Cartwright-Hatton et al., 2003	Community (*N* = 110)	8–11	Modified Speech task *speech delivered to camera*	Emotion Non-verbal Behavior Quality of speech	Performance Questionnaire: Global impression (*n* = 3 codes); micro-behaviors (*n* = 3 codes); nervous behaviors (*n* = 2 codes) (Internal consistency: α = 0.82 for the total score; Inter-rater reliability: *r* = 0.91)
Conelea et al., 2014	Clinical *Tic and co-occurring anxiety disorder* (*N* = 8)	8–12	Speech Task	Other (tics)	Frequency of tics (Inter-rater reliability = 77% agreement)
De Veld et al., 2014	Community (*N* = 140)	9–11	TSST-C	Non-verbal Behavior	Gaze aversion. (Inter-rater reliability: Cohen's kappa = 0.80)
Edmiston et al., 2017	Clinical *Autism Spectrum Disorders* (*N* = 28) *Tic Disorders* (*N* = 18)	*Autism Spectrum Disorders* *M* = 14.80 *Tic Disorders M* = 14.99 (Age ranges not provided)	TSST-C	Non-verbal Behavior	Displacement behaviors (i.e., face contact, repetitive motion with fingers or hands, “grooming” to enhance appearance, and lip movement); fidgeting; smiling (Inter-rater reliability: Cohen's kappa = 0.80)
Essau et al., 2014	School (*N* = 61) *- Referred by teachers for anxiety*	8–10	Modified speech task *Speech delivered to a group*	Emotion Quality of speech	Performance Questionnaire: Global impression (*n* = 3 codes); micro-behaviors (*n* = 3 codes); nervous behaviors (*n* = 2 codes) (Reliability not reported) Behavioral Signs of anxiety Scale Total score and 11 Unique codes: Nail-biting; Lip-licking; Mouth-touching; Sucking/chewing; Lip contortions; Lip biting; Hand movement to face; Hand movement to body; Hand movement to other; Leg movement (Reliability not reported)
Jansen et al., 2000	Clinical *Multiple Complex Developmental Disorder* (*N* = 10) Control (*N* = 12)	9–10	Modified speech task *Judges behind a mirror*	Quality of speech	Amount of time speaking; number of prompts (Reliability not reported)
Jansen et al., 2003	Clinical *Multiple Complex Developmental Disorder* (*N* = 10) *Autism* (*N* = 10) Control (*N* = 12)	9–10	Modified speech task *Judges behind a mirror*	Quality of speech	Amount of time speaking; number of prompts (Reliability not reported)
Jordan, 2008 (Dissertation)	Community (*N* = 362) *Divided into: socially phobic* (*n* = 78), *Socially anxious* (*n* = 60), *Non-anxious* (*n* = 203)	13–17	Speech task	Emotion Quality of speech Other (social skills)	Speech Rating Sheet: Anxiety; Social skills; Self-consciousness; Assertiveness; Friendliness; Attractiveness (Inter-rater reliability type unknown = 0.88 for anxiety, 0.86 for self-consciousness, 0.86 for social skill, and 0.84 for assertiveness)
Kertes et al., 2017	Community (*N* = 101)	7–12	TSST-C	Other (dyadic)	Dog proximity seeking: proportion of time dog stayed within child's reach; frequency of dog placing head in physical contact with the child; proportion of time the dog stayed in non-petting physical contact Child-solicited petting: frequency of commands from child to the dog; duration the child pet the dog (Inter-rater reliability: ICCs = 0.71–0.96)
Kramer et al., 2011	Clinical (*N* = 35) *Social Phobia* Control group (*N* = 35)	8–12	TSST-C	Emotion Non-verbal Behavior Quality of speech	Performance Questionnaire: Global impression (3 codes); micro-behaviors (3 codes); nervous behaviors (2 codes) (Internal consistency: α = 0.77 for global impression, 0.31 for nervous behaviors, 0.36 for micro-behaviors)
Lanteigne, 2011 (Master's thesis)	Community (*N* = 138)	12–16	Modified speech task *Speech delivered to camera*	Non-verbal Behavior	Self-Conscious Affect Code II (SCAC2) score comprising 8 domains: Body tension; facial tension; stillness; fidgeting; nervous positive affect; hiding, or avoiding; verbal certainty; silence (Inter-rater reliability: Cohen's Kappa > 0.65)
Lau, 2017 (Dissertation)	Mixed *Social Anxiety Disorder* (*n* = 34) *Non-anxious control* (*n* = 34)	8–14	Speech task	Non-verbal Behavior Quality of speech	Perception of Performance Questionnaires (POP-External Observer): Performance; How much the committee liked the speech (Internal consistency: α = 0.99) Social Performance Rating Scale (SPRS): Eye contact; Vocal quality; Discomfort; Speech flow. (Reliability not reported)
Lievesley, 2014 (Dissertation)	Clinical *Chronic fatigue syndrome* (*N* = 62) *Asthma* (*N* = 31) Control (*N* = 78)	11–18	Modified speech task *Speech delivered to the experimenter and video-camera*	Non-verbal Behavior Quality of speech	Speech Evaluation Questionnaire: Total Score (17 codes) Unique codes include: Friendly; Awkward; Relaxed; Embarrassed; Attractive; Nervous; Easy to understand; Blushing; Interesting; Stuttered or stammered; Confident; Left gaps in speech; Funny; Hands shaking; Uncomfortable; Clear voice; Avoid looking at camera (Reliability not reported)
Lozoff et al., 2014	Community (*N* = 1032)	10–11	Speech task	Emotion Non-verbal Behavior	Child self-confidence; child nervousness; smiling (frequency and latency to child's first smile); laughing; fidgeting; number of examiner prompts (Reliability not reported)
Miers et al., 2009	Community (*N* = 136)	9–17		Emotion Other (social skills)	Performance Questionnaire (modified scoring): social skills and nervousness (Internal consistency: α = 0.63 for social skills and 0.70 for nervousness)
Niekerk et al., 2017	Community (*N* = 141)	8–13	Modified speech task *Speech delivered to camera*	Emotion Other (social skills)	Performance Questionnaire (modified scoring): social skills and nervousness (Internal consistency: α = 0.71 for social skills and 0.61 for nervousness)
Oppenheimer et al., 2016	Clinical (*N* = 86) *Post-completion of anxiety treatment*	9–14	Modified speech task *Speech delivered to camera*	Other (dyadic)	Living in Family Environments coding scheme: Parent positive interpersonal scores, parent aggressive interpersonal scores, parent anxious affect (Inter-rater reliability: ICCs = 0.74 for anxious affect, 0.76 for positive internpersonal behavior, and 0.84 for aggressive interpersonal behavior)
Panjwani et al., 2016	Community (*N* = 200)	14–18	TSST-C	Emotion	Expression of happiness, sadness, anger, anxiety, contempt, shame/embarrassment coded using vocal, facial, and postural cues (Inter-rater reliability: Cohen's Kappa = 0.52 for emotions, 0.78 for happiness, 0.42 for sadness, 0.67 for anger, 0.35 for anxiety, 0.37 for contempt, and 0.63 for shame)
Pezdek and Salim, 2011	Community (*N* = 73)	14–18	Speech task	Non-verbal Behavior Quality of speech	Social Performance Rating Scale (SPRS): Total score (sum of 5 codes): Gaze; vocal quality; speech length; discomfort; conversation flow (Internal consistency: α = 0.72 for the total score; Inter-rater reliability: Cohen's Kappa = 0.86)
Rith-Najarian et al., 2014	Community (*N* = 79)	13–17	TSST-C	Non-verbal Behavior Quality of speech	Evaluated Speech Performance Measure (ESPM): Total performance score (sum of 11 codes: Number of explaining gestures to enhance the speech; volume of participant's voice; number of positive facial expressions displayed; number of negative facial expressions displayed; speech continuity (pausing or stopping); tension in the body; degree of closed posture; fidgety behavior; speech organization; confidence of presentation; quality of speech content (Internal consistency: α = 0.84 for the total score; Inter-rater reliability: Cohen's Kappa = 0.86–0.95)
Roth and Herzberg, 2017	Community (*N* = 112)	15–19	Modified speech task *Speech delivered to camera* *Participants asked to recount a specialized text*	Non-verbal behavior	Frequency of stress reactions -total of 17 unique codes within 4 categories: gaze, speech, posture/orienting, and self-manipulation. (Inter-rater reliability: *r* = 0.85) Speech Performance -amount of text reproduced (Inter-rater reliability: *r* = 0.72)
Wedl et al., 2016	Community (*N* = 19 boys)	7–11	TSST-C	Other (dyadic)	Percentage of time of the following: Physical contact with the dog; playing with the dog; talking to the dog; talking to investigator or dog-handler (Inter-rater reliability type unknown = 0.91% )

*N, sample size; TSST-C, Trier Social Stress Test for Children (with speech and arithmetic tasks); Speech Task, only the speech portion of the TSST-C; Modified speech task, only the speech portion of the TSST-C but with some modifications; PTSD, Post-traumatic Stress Disorder; M, mean*.

### Primary Coding Approaches

#### Non-verbal Behavior (*n* = 14)

Several of the studies reviewed coded non-verbal behaviors, with 14 out of 29 of the studies reporting at least one behavioral code (i.e., gaze, body movement, facial expression, and/or global non-verbal constructs). In terms of the types of non-verbal behaviors assessed, one master's thesis (Lanteigne, 2011), two dissertations (Lievesley, 2014; Lau, 2017), and six articles examined gaze or eye contact with the person(s) being spoken to or the camera (Cartwright-Hatton et al., 2003; Kramer et al., 2011; Pezdek and Salim, 2011; De Veld et al., 2014; Blöte et al., 2015; Roth and Herzberg, 2017). One master's thesis (Lanteigne, 2011) and six of the 29 articles investigated both body movement and facial expressions (Cartwright-Hatton et al., 2003; Kramer et al., 2011; Lozoff et al., 2014; Rith-Najarian et al., 2014; Burkholder et al., 2016; Edmiston et al., 2017). An additional dissertation (Lievesley, 2014) and article (Roth and Herzberg, 2017) coded body movement without also coding facial expressions.

##### Gaze

In two of the articles (Cartwright-Hatton et al., 2003; Kramer et al., 2011), coders used a global, 4-point Likert scale to assess the extent to which youth looked at the camera or person they were speaking to (ranging from “*not very much*” to “*very much*”). Cartwright-Hatton et al. (2003) coded speech tasks delivered by children aged 8–11 sampled from the community. In contrast, Kramer et al. (2011) examined gaze in a clinical sample of youth, aged 8–12, with social phobia and a control group delivering the TSST-C. Despite differences in samples and methods, both sets of authors analyzed this gaze code as part of the more comprehensive micro-behaviors subscale of the Performance Questionnaire-Observer version (PQ-O; Cartwright-Hatton et al., 2003). Kramer et al. (2011) noted that even though the PQ-O exhibits adequate internal consistency (α = 0.77), the micro-behaviors subscale specifically exhibited low reliability (α = 0.36). In both studies, the authors reported good inter-rater reliabilities for the overall PQ-O (ICCs = 0.71 and 0.91 for Kramer et al., 2011 and Cartwright-Hatton et al., 2003, respectively). Inter-rater reliability for the micro-behaviors subscale specifically was not provided, so it is unclear whether this subscale would hold up, given the poor internal consistency previously reported.

Pezdek and Salim (2011) and Lau (2017) assessed gaze on a global Likert scale (ranging from 1 = “*very poor, highly anxious*” to 5 = “*very good, less anxious*”) using the Social Performance Rating Scale (SPRS; Fydrich et al., 1998). Pezdek and Salim combined gaze ratings with the ratings of four other dimensions (i.e., vocal quality, speech length, discomfort, and conversation flow) to create a SPRS composite score, which in their study exhibited high inter-rater reliability (κ = 0.86). Blöte et al. (2015) also used a 4-point Likert scale to measure the extent to which participants “looked at the audience.” In contrast to Pezdek and Salim, who used gaze to assess overall speech performance, Blöte et al included gaze as part of an agitation subscale on the Speech Performance Observation Scale for Youth.

Lievesley (2014) used the Speech Evaluation Questionnaire (SEQ; Harvey et al., 2000) to rate videos of participants giving a 3-min speech. The SEQ was originally developed as a self-report measure, yet it was adapted here for use by independent observers. Coders viewed video-recordings and coded for gaze aversion using a global, 10-point Likert scale, ranging from “*not at all*” to “*extremely”* (i.e., looking away from the camera), which was just one of 17 total codes on the SEQ. The sample included adolescents, aged 11–18, with chronic fatigue syndrome and asthma as well as a control group, who completed a modified speech task. No reliability information was provided. The same questionnaire was given to the participants to self-evaluate their performance and overall SEQ scores were calculated separately for the observer- and self-report. The self and observer composite SEQ scores were used to calculate and analyze discrepancy scores (i.e., how well the participant thought they did on the task subtracted from the independent observer score).

In a master's thesis, Lanteigne (2011) used The Self-Conscious Affect Code II (SCAC-II; Lanteigne et al., 2010) to code gaze as part of a broader construct labeled “hiding and avoiding” eye contact. Hiding/avoiding eye contact was coded dichotomously (i.e., yes or no depending on whether hiding/avoidance was present) for each minute interval of the task. Specifically, a code of “*none*” for hiding/avoiding reflected eye contact with the experimenter/camera, looking generally in a forward direction, or looking in any direction within the general vicinity of the experimenter (e.g., up, side, slightly down). Alternatively, hiding/avoiding would be noted as “*yes*” if the youth had their eyes closed for at least 3 s, turned their eyes and head away from the experimenter/camera, or used a hand to hide the face. The hiding/avoiding construct was combined with other affect cues and analyzed as part of a weighted self-conscious expression score. Inter-rater reliability for SCAC-II was adequate at 0.65 (kappa).

Roth and Herzberg (2017) developed a coding scheme that assessed for 17 verbal and non-verbal markers of “stress reactions” during a speech task delivered by German high school students aged 15–19. A composite variable of gaze was included as one of the 17 codes. While the full coding system was not provided, the authors described examples of gaze as blinking often and changes in viewing direction. Videos of the 3-min speech tasks were coded by tallying the number of occurrences of the 17 codes for each 30-s interval (i.e., an event-based coding approach). The authors combined the tallies to analyze the total number of stress reactions observed during the speech. Interrater reliability for the coding in its entirety was good (ICC = 0.85).

In another study with a community sample of children aged 9–11, a 2-s interval coding approach was used to code whether youth looked at vs. away from the confederates and camera (De Veld et al., 2014). No composite scores were created. The authors reported high inter-rater reliability (κ = 0.80).

Taken together, the reviewed articles, master's thesis, and dissertations indicate that gaze was conceptualized differently based on the research team. In some studies, gaze was part of a larger construct reflecting self-consciousness (Lanteigne, 2011) or anxiety/stress (Roth and Herzberg, 2017). In other research, gaze was used as a standalone construct (De Veld et al., 2014). Gaze was approached from both dichotomous (e.g., whether gaze was averted vs. not) and continuous perspectives (e.g., Likert scale on the SEQ, proportion of time gaze was averted). It is notable that gaze was important to researchers regardless of whether the sample was a clinical or community sample or if the youth were children or adolescents.

##### Body movement

Other non-verbal behaviors also emerged. One master's thesis (Lanteigne, 2011) and seven of the 29 articles investigated both body movement and facial expressions (Cartwright-Hatton et al., 2003; Kramer et al., 2011; Lozoff et al., 2014; Rith-Najarian et al., 2014; Blöte et al., 2015; Burkholder et al., 2016; Edmiston et al., 2017). One additional dissertation (Lievesley, 2014) and article (Roth and Herzberg, 2017) coded body movement without also coding facial expressions. Body movement (sometimes referred to as “bodily expression” by the authors) included a very wide range of codes and behaviors such as: fidgeting, self-touch (e.g., scratching oneself), hand contact with the face, wary gait, muscular tension, facial tension, lip movement unrelated to speech (e.g., lip biting or licking), deep sighs, fiddling with or adjusting hair or clothing, blushing, hand-tapping, hand-shaking, repetitive movements of fingers, or hands, diminished activity level, posturing/orienting (e.g., defensive stance, changing posture often), shuffling feet, body swaying, stillness, shaking, stiffness, leaving the room, and taking a break from the assessment.

The eight papers varied in their approach to coding body movements. Rith-Najarian et al. (2014), for instance, developed the Evaluated Speech Performance Measure (ESPM) to code the TSST speech task delivered by a community sample of adolescents aged 13–17. The ESPM generates a composite performance score comprised of 11 items, including three items related to body presentation, that were each rated on a 5-point Likert scale (i.e., extent of body tension, closed posture, and fidgety behavior). Inter-rater reliabilities across pairs of coders were good for the ESPM overall (0.86–0.95). In contrast to this global performance approach, Burkholder et al. (2016) developed the Child and Adolescent Stress and Emotion Scale (CASES) to code the TSST-C for markers of anxiety with community participants aged 9–10 and 15–16. Their coding scheme contains a subscale aimed at measuring anxiety-related bodily expressions. Examples of codes, which were rated on a 4-point Likert scale, include muscular tension, defensive body posture, fidgeting, and self-touch. The bodily expression score was combined with a vocal and a facial expressions of anxiety score to create an overall anxiety expression composite which exhibited good inter-rater reliability (ICC = 0.80).

Some authors coded frequency of body movement (Lozoff et al., 2014; Roth and Herzberg, 2017), whereas others used binary codes for denoting presence or absence of each behavior for each moment of the stressful task (e.g., body tension cues, facial tension, stillness vs. movement, fidgeting, smiling or laughing; Lanteigne, 2011). In some studies (Blöte et al., 2015), investigators examined various movements as part of a broader construct (e.g., the agitation subscale of the Speech Performance Observation Scale for Youth). Other investigators (e.g., Edmiston et al., 2017) coded and analyzed the frequency of distinct body movements including face touch, lip press or bite, hand fumble, and grooming of adjusting hair or clothing. They reported high interrater reliability using Cohen's Kappa (κ = 0.80).

Lastly, blushing was coded by two articles (Cartwright-Hatton et al., 2003; Kramer et al., 2011) and one dissertation (Lievesley, 2014). Both Cartwright-Hatton et al. and Kramer et al. used a 4-point Likert scale to code the extent to which youth blushed (ranging from “*not very much*” to “*very much*”). This blushing code was part of a more comprehensive nervous behaviors subscale on the PQ-O. In Lievesley (2014), the blushing construct, which was coded on a 10-point Likert scale, was part of the SEQ overall score (combined with hands shaking).

##### Other non-verbal

In a dissertation, Benoit (2013) coded four global constructs (avoidance, non-compliance, engagement, and anxiety) that likely were comprised of codes specific to body movements. However, this information was not provided, so it is unclear exactly how these behaviors were coded. Intraclass correlation coefficients were reported for each scale separately (non-compliance = 0.40, avoidance = 0.89, and engagement = 0.83). The scale scores were used to calculate an average (or total count), but because of the low reliability of the non-compliance scale, that scale was not used in the analyses.

##### Facial expressions

Facial expressions were coded in one master's thesis (Lanteigne, 2011) and six articles (Cartwright-Hatton et al., 2003; Kramer et al., 2011; Lozoff et al., 2014; Rith-Najarian et al., 2014; Burkholder et al., 2016; Edmiston et al., 2017). The range of facial expressions included wincing, furrowed brow, widened eyes, tearfulness, crying, frowning, (inappropriate) smiling, and laughing. Within facial expressions, smiling was the most frequent behavior coded. However, authors varied in how they approached this construct. For instance, Cartwright-Hatton et al. (2003) and Kramer et al. (2011) used the PQ-O, which assesses, on a 4-point Likert scale, the extent to which the youth smiled during the speech task. Along with gaze, the smile code is part of the micro-behaviors subscale of the PQ-O, which has shown poor internal consistency. The master's thesis (Lanteigne, 2011) coded for smiling and laughing using a dichotomous approach (i.e., for presence vs. absence) and included this code as part of a larger nervous or anxiety affect construct (see Emotions section for more details). Other coding approaches to coding smiling were event-based (Lozoff et al., 2014; Edmiston et al., 2017), and one group coded the latency to the first smile (Lozoff et al., 2014).

Other facial expressions were also noted. For instance, Burkholder et al. (2016) used CASES to assess, using a 4-point Likert scale, the intensity of facial signs of anxiety (e.g., wincing, widened eyes, raised or furrowed brow, tearfulness, and distinctive frown) from none to severe. Similarly, the ESPM (Rith-Najarian et al., 2014) includes one facial expression item—the number of positive and negative facial expressions the participant displayed—which is rated on a 5-point Likert scale. This code is part of a broader performance score.

#### Emotion (*n* = 12)

Of the studies reviewed, 12 out of 29, incorporated measures of emotion or affect. A myriad of different specific emotional expressions was assessed, but all generally served as indicators of anxiety induced by the speech task (e.g., Cartwright-Hatton et al., 2003; Jordan, 2008; Miers et al., 2009; Kramer et al., 2011; Benoit, 2013; Essau et al., 2014; Lievesley, 2014; Lozoff et al., 2014; Blöte et al., 2015; Burkholder et al., 2016; Panjwani et al., 2016; Borelli et al., 2017; Niekerk et al., 2017). Several of the coding approaches aimed to quantify levels of anxiety or nervousness by coding non-verbal expressions of anxiety (i.e., fidgeting, self-touch, wary gait, muscular tension, hand-tapping, diminished activity level, defensive posturing, body swaying, hands shaking, stiffness, leaving the room; rapid or repetitive movement of the hands or torso; e.g., Lievesley, 2014; Lozoff et al., 2014; Burkholder et al., 2016; Panjwani et al., 2016; Borelli et al., 2017) and nervous facial expressions (i.e., wincing, furrowed brow, widened eyes, tearfulness, frowning, smiling, lip-licking; laughing; rapid or repetitive movement of the eyes or mouth; e.g., Essau et al., 2014; Lozoff et al., 2014; Burkholder et al., 2016; Panjwani et al., 2016; Borelli et al., 2017). Some of these were described previously in the Non-verbal Behavior section. Studies varied in how they measured emotional expression, with some studies using Likert scales (e.g., Lozoff et al., 2014; Burkholder et al., 2016; Panjwani et al., 2016; Borelli et al., 2017). For instance, Borelli et al. (2017) coded school-aged children's anxious non-verbal cues (e.g., rapid or repetitive movement in the hands, eyes, mouth and torso) utilizing a 7-point Likert scale. Burkholder et al. (2016) and Panjwani et al. (2016) utilized 4-point Likert scales and 3-point Likert scales, respectively to code more comprehensive sets of emotional behavior, such as body expressions (Burkholder et al., 2016), facial expression (Burkholder et al., 2016; Panjwani et al., 2016), and posture (Panjwani et al., 2016). Others, like Lozoff et al. (2014) coded a single discrete behavior, like fidgeting, on a 4-point scale. While these studies used Likert scales, others used frequencies, counting the number of occurrences of a given emotional expression per observation segment, and then calculating the total percentage of observation time the expression was present. As an example, Essau et al. (2014) coded whether 10 behavioral signs of anxiety were present or absent during 30-s intervals, and then mean scores were calculated across time intervals.

Five out of 12 studies included codes to measure anxious speech. For instance, Burkholder et al. (2016) focused on speech content by recording how often a person's speech included anxious subject material. Other studies (e.g., Cartwright-Hatton et al., 2003; Kramer et al., 2011; Lievesley, 2014; Panjwani et al., 2016) focused on vocal expressions of anxiety, such as vocal quaking, stumbling over words or stuttering. Often the codes were kept separate, although other times they were combined to create an overall anxiety expression score. For instance, both Burkholder et al. (2016) and Panjwani et al. (2016) averaged several distinct codes (e.g., facial expressions, body expressions/posture, vocal qualities) to form composites.

It is noteworthy that six of the 12 studies included global measures of emotion. For instance, Lozoff et al. (2014) and Kramer et al. (2011) had coders rate children's overall nervousness using a 4-point Likert scale. Likert scales were also used by Benoit (2013), Jordan (2008), Essau et al. (2014), and Lievesley (2014) to measure emotion more globally. For instance, coders rated on a 10-point Likert scale how nervous he/she came across (Lievesley, 2014), on a 7-point Likert scale how anxious the participant was during the speech (Jordan, 2008), how upset he/she was during the task (Benoit, 2013), or on a 4-point Likert scale how nervous he/she looked (Cartwright-Hatton et al., 2003; Essau et al., 2014). Despite these coding systems not being behaviorally specific, they seemed to demonstrate adequate interrater reliability (Interrater reliability ranged from 0.87 to 0.88; Jordan, 2008).

While all studies coded anxiety, Panjwani et al. (2016) also coded emotional expressions of happiness, sadness, anger, contempt and shame using vocal, facial and postural cues. Emotional expressions were coded on a 3-point Likert scale, with a 3 indicating that an emotion was expressed in multiple channels (i.e., vocal, facial, and/or postural). Given that sadness and contempt occurred infrequently during the TSST, the reliability for these emotion codings were low. However, happiness, anger, and shame showed Kappa values that would be considered moderate in the literature (ranging from κ = 0.63–0.78).

#### Speech Performance and Quality (*n* = 10)

Several of the published studies and dissertations were interested in measuring overall speech quality or performance. In some studies, a coding scheme was developed by the authors specifically for their study (e.g., the Evaluated Speech Performance Measure; Rith-Najarian et al., 2014). Other studies employed already-existing coding paradigms such as the Performance Questionnaire (Kramer et al., 2011; Essau et al., 2014), the Perception of Performance Questionnaire—External Observer (Lau, 2017), and the Social Performance Rating Scale (Pezdek and Salim, 2011; Lau, 2017). Studies varied in the number of unique codes that comprised the overall performance score. Two studies by the same authors (Jansen et al., 2000, 2003), for example, examined performance in a 5-min speech task in a group of 10 children (mean age: 9 years) with multiple complex developmental disorders and a group of 12 healthy controls. The authors measured performance using two specific codes—the amount of time the child was talking and the number of prompts required by the experimenter. Each of these codes were used as outcome variables. No reliability information was provided.

One dissertation (Jordan, 2008) and two published studies (Cartwright-Hatton et al., 2003; Rith-Najarian et al., 2014) developed coding approaches specifically for their study's research questions. In Jordan (2008), the author used a 6-item Speech Rating Sheet (which is included as an appendix in the dissertation) to rate, using a 7-point Likert scale, how anxious, socially-skilled, self-conscious, assertive, friendly, and attractive the adolescent appeared during a 10-min speech task. Both the social skills and assertiveness ratings exhibited good inter-rater reliability (0.84–0.87), and these codes were used as unique outcome variables, measuring distinct performance qualities.

Cartwright-Hatton et al. (2003) developed the Performance Questionnaire-observer rating (reviewed previously), which has been used subsequently by several other authors (Kramer et al., 2011; Essau et al., 2014). The PQ-O offers a global impression subscale comprised of three items: How friendly did the child look? How clever did the child look? How good was the child's speech? Each of these items are rated by observers using a 4-point Likert scale. In our review, the PQ-O was used in research with the TSST-C and in research using a modified speech task. The PQ-O was used with children ranging in age from 8 to 13. As mentioned previously, the overall PQ-O exhibits good inter-rater reliability (*r* = 0.91) and internal consistency (α = 0.82), but studies that have reported the reliability coefficients for each of the three components of the PQ-O have not met the same standards (α = 0.31, 0.36, and 0.77 for the nervous behaviors, micro-behaviors, and global impression, respectively). One additional study used the PQ-O (Niekerk et al., 2017), but the authors did not examine the global impression subscale. This study is therefore discussed below under *Other Coding Approaches: Social skills*.

Rith-Najarian et al. (2014) also developed their own coding scheme, the Evaluated Speech Performance Measure (ESPM). The ESPM, unlike the Jordan (2008) Speech Rating Sheet and the PQ-O, offers a composite performance score taking into account 11 performance qualities [i.e., explaining gestures, smiling, making faces/grimacing, voice volume, body tenseness, posture (open vs. closed), fidgeting, long pauses, how thought-out the speech was, how comfortable the participant appeared, and overall speech quality]. The first 8 codes are rated on a 5-point Likert scale, and the last 3 codes are rated on a 7-point Likert scale. Scores on the ESPM can range from 11 to 61, thus allowing for considerable variability in performance. Inter-rater reliabilities for all codes were good (0.84–0.95).

One published study (Pezdek and Salim, 2011) used the Social Performance Rating Scale, developed by Fydrich et al. (1998), in a sample of adolescents aged 14–18 completing a speech task. This coding scheme was originally designed to evaluate performance during a conversational speech task, yet it was applied here to test post-treatment differences in performance anxiety between a treatment (activating autobiographical memories) vs. a control group. It includes five dimensions—gaze, vocal quality, speech length, discomfort, and conversation flow—which are rated on a 5-point Likert scale. Overall performance is evaluated by summing scores on these five dimensions, with good internal consistency (α = 0.72) and inter-rater reliability (*r* = 0.79–0.82). Lau (2017) also used the Social Performance Rating Scale, but in a sample of younger children aged 8–14. In this study, children with Social Anxiety Disorder and non-anxious, healthy controls were compared on their performance on the TSST speech delivery.

One dissertation (Lievesley, 2014) used the Speech Evaluation Questionnaire (Harvey et al., 2000), which was originally developed as a self-report questionnaire used for the self-evaluation of 17 distinct qualities (e.g., friendliness, embarrassment, confidence, stuttering). In Lievesley (2014), the author used this questionnaire to also provide an objective rating of overall performance. Two coders rated video-recordings of modified speech tasks delivered by adolescents aged 11–18, and an average of the two coders' total scores on the measure was used as the outcome variable. No reliability information was provided.

Finally, one dissertation (Lau, 2017) used the Perception of Performance Questionnaire—External Observer (PPO) to assess performance in youth aged 8–14 with Social Anxiety Disorder and non-anxious controls. The PPO only contains two questions, which raters rate on a 0–10 Likert scale: “How do you think the child performed on the speech?” and “How much do you think the committee liked the child's speech?” Only internal consistency was reported (α = 0.99), so it is unclear how reliable independent raters would be on this measure.

#### Other Coding Approaches

##### Dyadic (n = 5)

Five of the 29 articles found included a dyadic coding approach (Beetz et al., 2011, 2012; Oppenheimer et al., 2016; Wedl et al., 2016; Kertes et al., 2017). In these studies, 7-to-11-years-old boys completed the TSST-C with one of three social support conditions (presence of dog, toy dog, or friendly female confederate). The authors coded for 49 unique variables, several of which likely overlap with previously-discussed categories above. Nonetheless, attachment-related variables included: seeking physical contact (e.g., body contact, stroking/petting) and seeking social contact with the “social supporter” (e.g., talking to the supporter). Variables were coded on the basis of frequency (occurrence per minute of observation) and duration (% of observation time during which the behavior occurred) using the Noldus Observer. Only one of the four published studies was from a distinct research group (Kertes et al., 2017). Kertes et al. used the TSST-C with a sample of 7–12-years-old children. Five behaviors were coded throughout the TSST-C, which were all focused on the dyadic interaction between the child participant and the pet dog (e.g., duration of time the dyad was in contact, number of solicitations given by the child to the dog). Intraclass correlations ranged from 0.71 to 0.96.

One other article approached the speech task from a dyadic perspective (Oppenheimer et al., 2016). In their study, 86 clinical youth aged 9–14 and their parents participated in a modified speech task (modified because the speech was delivered to a camera rather than to live judges). Three parent variables were coded during this interaction using the Living in Family Environments coding system (LIFE; Hops et al., 1995a,b). These variables included parent positive interpersonal scores, parent aggressive interpersonal scores, and parent anxious affect. The coding scheme takes an event-based, microanalytic approach, and yields a frequency score, which the authors converted into a rate per minute score with good intraclass correlation coefficients (ICCs = 0.74–0.84).

##### Tics (n = 2)

Two of the 29 works measured the frequency of tics during the speech delivery (Conelea et al., 2014; Buse et al., 2016). Both studies used a clinical sample of youths with tic disorders (Buse et al., 2016) and comorbid tic and anxiety disorders (Conelea et al., 2014). Sample sizes were generally small (Ns = 31 and 8, respectively), and youth ranged in age from 7 to 17. Reliability was only reported in one of the studies and fell in the good range (77% agreement).

##### Social skills (n = 3)

Two published study (Miers et al., 2009; Niekerk et al., 2017) and one dissertation (Jordan, 2008) examined levels of social skills during speech delivery. Miers et al. (2009) and Niekerk et al. (2017) used the PQ-O with modified scoring. This scoring approach yields two rather than three subscales (i.e., social skills and nervousness; Miers et al., 2009). In Miers et al. (2009), the authors incorporated two additional questions to the PQ-O including: “How much did the speaker look at the audience?” and “Did the speaker have blotches in his/her face?” Both questions correspond to the social skills subscale. Inter-rater reliability for this subscale was excellent at 0.94 (intraclass correlation). In Jordan (2008), one item on the Speech Rating Sheet assesses social skills (i.e., “How socially-skilled did you think the participant was during the speech?”), which is rated by observers using a 7-point Likert scale (inter-rater reliability = 0.86). However, several other items might also offer information about social skills, such as how friendly the participant appears.

## Discussion

The current review examined behavioral observation coding approaches to the Trier Social Stress Test for Children (TSST-C) and modified versions of the TSST-C—i.e., speech tasks. In total, 29 published articles, dissertations, and master's theses were identified with a wide range of approaches to coding the TSST-C. The take-home finding from the current review is that there is no standard way to code the TSST-C, which appears to stem from the uniqueness of investigators' research questions and sample demographics. This lack of standardization prohibits any comparisons between studies and samples. Below, we discuss relevant implications and offer suggestions for future research.

The fact that 24 unique coding approaches were identified speaks to investigators' interest in quantifying observed behaviors/performance during this task. Some approaches were focused on just a few codes while other approaches included more comprehensive coding schemes with subscales or composites. In general, however, most coding schemes were interested in both behavioral and emotional aspects of the speech delivery, making the distinction between behavior and emotion almost arbitrary. For instance, fidgeting is a behavior that was coded as part of a broader nervousness (e.g., Self-Conscious Affect Code II; Lanteigne, 2011) and performance construct (e.g., ESPM; Rith-Najarian et al., 2014). Quality of speech or overall speech performance appeared to be coded less frequently than expected (10 studies), suggesting that investigators may be interested in more nuanced constructs. As mentioned previously, only two coding approaches were included more than once, the Performance Questionnaire—Observer version and the coding scheme used by Beetz et al. (2011, 2012). The PQ-O was used in five articles, making it the most widely-used coding scheme.

There was also significant variability in how the constructs were measured. In some studies, for example, investigators measured constructs using a global rating (i.e., a Likert scale of the extent to which the construct was present; e.g., Lozoff et al., 2014). Likert scales were also used to rate constructs on an interval basis—i.e., one rating for every 1-min interval of the speech delivery. In contrast, other studies used an event-based approach where they coded the construct every time it occurred (i.e., a frequency count of the behavior; e.g., Edmiston et al., 2017). The global coding approach was the most widely-used, which may be because event-based approaches are more intensive because they require more precision. Event-based coding also raises important questions about how inter-rater reliability should be calculated. Is it sufficient to calculate inter-rater reliability on the total frequency count of the code, or is agreement only considered when the coding of an event occurs around the same time for both coders? These questions make event-based coding more complex. Investigators may also feel that event-based coding is too precise and structured, and does not allow for rater judgements that may be included in a global code.

### Limitations of Existing Coding Schemes

One potential limitation is that the coding schemes reviewed were not all developed for coding the TSST-C. Several of the coding approaches, including the PQ-O, the Self-Conscious Affect Code II, the Speech Evaluation Questionnaire, and the Social Performance Rating Scale were developed for purposes other than coding the TSST-C and were later adapted. The Performance Questionnaire-Observer and the Speech Evaluation Questionnaire, for instance, were originally self-report measures, which were then modified for observer use. There is a clear benefit to this, which is that the two versions (i.e., self-report and observer versions) can be compared, contrasted, or combined into a multi-reporter composite score. The disadvantage is that there may be important constructs for which a self-rating might not be as accurate or informative. For example, it might be more difficult for a participant (vs. an observer) to rate how much he or she blushed during the speech. It may also be the case that some codes are no longer relevant once they are applied to the TSST-C. The Social Performance Rating Scale, for example, was originally developed to code conversational tasks. The applicability to the TSST-C in that case might be limited given that live judges are instructed to not converse with participants and to appear stoic. Part of the Speech Flow code involves rating the participant's ability to offer follow-up remarks in response to the individual they are conversing with. This does not apply during the TSST-C. In addition, a common modification to the TSST-C was the use of video-recording instead of live judges, which is likely to impact coding schemes that were developed based on conversational or dyadic exchanges.

Another potential limitation is that the coding approach (e.g., global, interval, event-based) guides the types of questions that can be addressed. Behavioral observation coding requires significant resources, especially when samples are large and when the approach is microanalytic and event-based. For some codes, a global rating may be sufficiently informative. Depending on the research questions, and on the structure of other variables of interest, a single global code may limit the conclusions that can be drawn about the TSST-C speech delivery. For instance, there is a great interest in measuring autonomic nervous system activation during the speech (Birkett, 2011). In some instances, ANS markers are examined minute-by-minute and then averaged (e.g., mean RSA during the 5-min speech delivery). In other instances, the investigator is interested in moment-by-moment changes in these constructs throughout the course of the speech, or in one sole moment—e.g., the first minute or the moment during which activation is at its peak. Global coding approaches cannot capture these nuances and cannot be compared to other measurements when the latter are examined in a more precise, event-based fashion. Global coding approaches cannot answer questions that are grounded in change throughout the speech delivery. Nonetheless, it is a fine balance between the global and more intensive interval and event-based approaches.

Reliability is an important factor when considering a coding scheme. In the current review, a majority of authors reported some sort of psychometric information (inter-rater reliability and internal consistency). However, these metrics used were not always ideal. In some instances, authors reported the inter-rater reliability for the scale in its entirety, even if subscales were used in the analyses. If specific subscales are used, then each subscale should also meet acceptable reliability cutoffs, and these coefficients should be reported. This was not always the case. In other instances, there was significant variability in inter-rater reliability reported by authors. Even though it was the most widely used approach, the three subscales of the PQ-O did not always meet acceptable reliability cutoffs, which is problematic for researchers seeking to implement this coding approach in their research. Articles mostly reported on the internal consistency of the three PQ-O subscales and on the inter-rater reliability of the overall PQ-O. There was no information about whether raters are reliable in their coding of the specific codes that make up the three subscales.

Finally, an important challenge to highlight is the balance between comprehensiveness and specificity. As we indicated in the review, investigators used approaches to suit their research questions and sample demographics. For instance, authors interested in measuring tic frequency during the TSST-C in a sample of youth with tic disorders may not be interested in other behavioral or affective codes. In such cases, authors are interested in a high level of specificity in their coding approach, but are perhaps not as focused on a high level of comprehensiveness. Two coding approaches in particular stood out as striking a balance between specificity and comprehensiveness. The study by Blöte et al. (2015) was one of the few studies that demonstrated comprehensiveness and specificity. Their coding scheme, the Speech Performance Observation Scale for Youth (SPOSY), was specifically developed to comprehensively assess behaviors displayed by anxious youth. Their unique approach of using “naïve” observers to generate observations of anxious youth led to the development of a coding approach that was highly relevant to this population of youth. Investigators interested in measuring anxiety during the speech task may wish to consider this coding scheme. Other approaches were focused on a comprehensive performance score. One such example is the ESPM, which yields a total score comprised of 11 unique codes, some of which are very objective (e.g., number of pauses longer than 5 s), and others require some level of judgment about the overall quality of the speech (e.g., “How well thought out was the speech?” or “How do you feel the participant did?”). These latter questions allow the observer to account for factors such as developmental level of the child. For example, developmental level is likely to impact how “thought out” or organized a speech can be. No other coding scheme combined this range of objective-subjective codes. In addition, the score range offered by the ESPM (11–61) is important for samples that may have wide-ranging performance abilities (e.g., samples with a wide age range).

### Recommendations for Future Research

1. There is a need for greater consistency and standardization in this subset of the behavioral observation literature. One goal of the current review was to take an initial step toward surveying the literature to determine what types of coding schemes investigators are using to code the TSST-C. There was little consistency, even within similar age groups or sample type (e.g., clinical samples of anxious youth). Greater consistency is needed to determine whether coding approaches remain reliable across samples and task modifications. The goal here would be to identity and adopt the most valid and reliable coding approaches to standardize the way investigators approach coding behavioral observations. Understandably, it may be that unique sample demographics call for specific coding approaches. Nonetheless, it is likely (and preferred) for samples similar in age and presenting problem to be coded in similar ways. This level of standardization and consistency would allow investigators to compare findings across studies.
2. Coding schemes should be readily available to investigators. Full coding schemes should be published as appendices, supplemental material, or even as standalone development and validation papers. In scientific writing, it is required to report on the measures used. Measurement approaches, such as behavioral observation coding, should be replicated precisely (assuming they exhibit good psychometric properties). This is very challenging when details regarding the actual codes, descriptors, coding approach, scaling, and training information is not reported. In this review, it was uncommon to have all of this information available within the article.
3. Coding schemes should be developed and tested using rigorous methods and data analytic approaches, much like methods required for the development and validation of questionnaires. We encourage authors to publish their coding schemes as validation papers with all the required information for investigators to determine whether the coding approach would apply to their research questions and sample. Relatedly, as indicated above, more information is needed about the inter-rater reliabilities of the specific subscales within the broader coding scheme. In this review, coding schemes were developed for student dissertations. The development of a coding scheme is a laborious process and stands to contribute in a significant way to the field. As such, students should consider publishing their development process as a standalone paper.
4. We encourage authors to be thoughtful and thorough when choosing a coding approach. Investigators should not simply use a coding scheme without understanding its properties (including psychometric properties) and the method(s) by which it was developed. It is tempting to search for “TSST-C and behavioral observations” and pick a coding scheme that is most widely-used or cited. This does not guarantee the best psychometric properties nor does it guarantee that the coding approach will be the best option for the proposed research questions.
5. Finally, if authors are seeking to use a well-validated behavioral observation coding scheme, we recommend the ESPM and the SPOSY. The ESPM is brief, applicable to non-clinical samples, and demonstrates good inter-rater reliability. The ESPM examines non-verbal behaviors and overall quality of the speech. The SPOSY was carefully developed and tested, and appears particularly useful to use with individuals with anxiety symptoms and disorders. It examines both emotion and non-verbal behavior, but does not appear to yield an overall speech quality score.

## Conclusion

The current review examined behavioral observation coding approaches for the TSST in child and adolescent samples. Findings should be interpreted within the study's limitations. For example, there were insufficient consistency in use of coding schemes across studies. This prevented us from conducting any meta-analytic analyses. Second, several articles did not report specific details about the subscales, or distinct components, of their coding paradigm. This made it challenging to more accurately evaluate the reliability and validity of the various components making up the coding scheme. Results of the review, including review limitations, highlight areas for future research and recommend that researchers exert caution in selecting coding paradigms for their research. Taken together, there are clear benefits to using behavioral observations to measure responses to the TSST. These responses are likely to provide insights into the effectiveness of the TSST in eliciting a stress response and also in measuring individual differences in response to stress.

## Author Contributions

KT conceived and designed the study and conducted the systematic search. JR-H and JH assisted in coding articles. KT, JR-H, and JH drafted the manuscript. All authors approved the final version of the manuscript for submission.

### Conflict of Interest Statement

The authors declare that the research was conducted in the absence of any commercial or financial relationships that could be construed as a potential conflict of interest.
